# Welfare implications on management strategies for rearing dairy calves: A systematic review. Part 1–feeding management

**DOI:** 10.3389/fvets.2023.1148823

**Published:** 2023-03-30

**Authors:** Patricia Carulla, Arantxa Villagrá, Fernando Estellés, Isabel Blanco-Penedo

**Affiliations:** ^1^Instituto de Ciencia y Tecnología Animal, Universitat Politècnica de València, Valencia, Spain; ^2^Centro de Tecnología Animal, Instituto Valenciano de Investigaciones Agrarias, CITA-IVIA, Polígono de La Esperanza, Segorbe, Castellón, Spain; ^3^Departamento de Ciencia Animal, Universidad de Lleida, Lleida, Spain; ^4^Department of Clinical Sciences, Swedish University of Agricultural Sciences, Uppsala, Sweden

**Keywords:** rearing calves, Holstein calves, welfare, feeding management, animal production, dairy sector

## Abstract

**Introduction:**

Calves are very susceptible to stress in the early stages of life, and it is necessary to ensure maximum welfare. Feeding management has been identified as a major risk factor for calf health and welfare at this stage. However, the management protocol for calf rearing and its impact on animal welfare is unclear. A systematic review of different management strategies for rearing dairy calves according to the three spheres of animal welfare was conducted using an electronic search strategy. In this review, management strategies were studied to identify scientific gaps, to know the welfare problems of these animals in order to prioritize actions and future research and to study the interpretive approach of this management from the three welfare spheres.

**Methods:**

A protocol was used to analyze and extract information from the studies. Of the 1,783 publications screened, only 351 met the inclusion criteria for the management or welfare of calves' items.

**Results:**

The publications identified in the search can be divided into two main groups feeding and socialization, based on the main topic of the publication. The main topics that emerged from the search in the feeding management group were milk replacer, colostrum, and weaning, divided into the three main areas of biological functioning and health, natural life and affective states or cognitive judgement.

**Discussion:**

The main issues to be addressed were the different types of feed consumed by animals from birth to weaning and the weaning management. It has been found that the most researched issues are colostrum and solid starter feed management. Unresolved issues were highlighted, such as the lack of a clear protocol for the administration of milk replacers to reduce hunger and the best management of weaning to reduce stress.

## 1. Introduction

One of the major challenges in livestock production's is to ensure animal welfare at all stages of rearing. In dairy cattle, calf rearing is one of the most challenging aspects of animal welfare and the second-highest variable cost after feeding (1). Furthermore, optimizing calf rearing has a massive impact on the future production of the cow, thus making it a key issue for welfare, production, and economic sustainability.

To ensure animal welfare, it is necessary to know how to assess it. There have been significant changes in the assessment of animal welfare in recent decades. The current scientific approach to animal welfare by science is not yet standardized. Although there is a scientific process and an increasing consumer demand for animal welfare, regulations are only focus on the basics (2, 3). One of the reasons for this lack of specific regulation may be the lack of consensus on the concept of animal welfare. In recent years, there has been an evolution from avoiding negative experiences to exploring positive experiences for animals, recognizing that good welfare, a “*good life*,” is not only about preventing negative states, but also about promoting positive experiences and emotional states (4–6). Positive animal welfare and its evaluation emphasizes resources valued by animals, positive emotions, and the natural behaviors that animals are motivated to perform (5).

**Figure 1:**
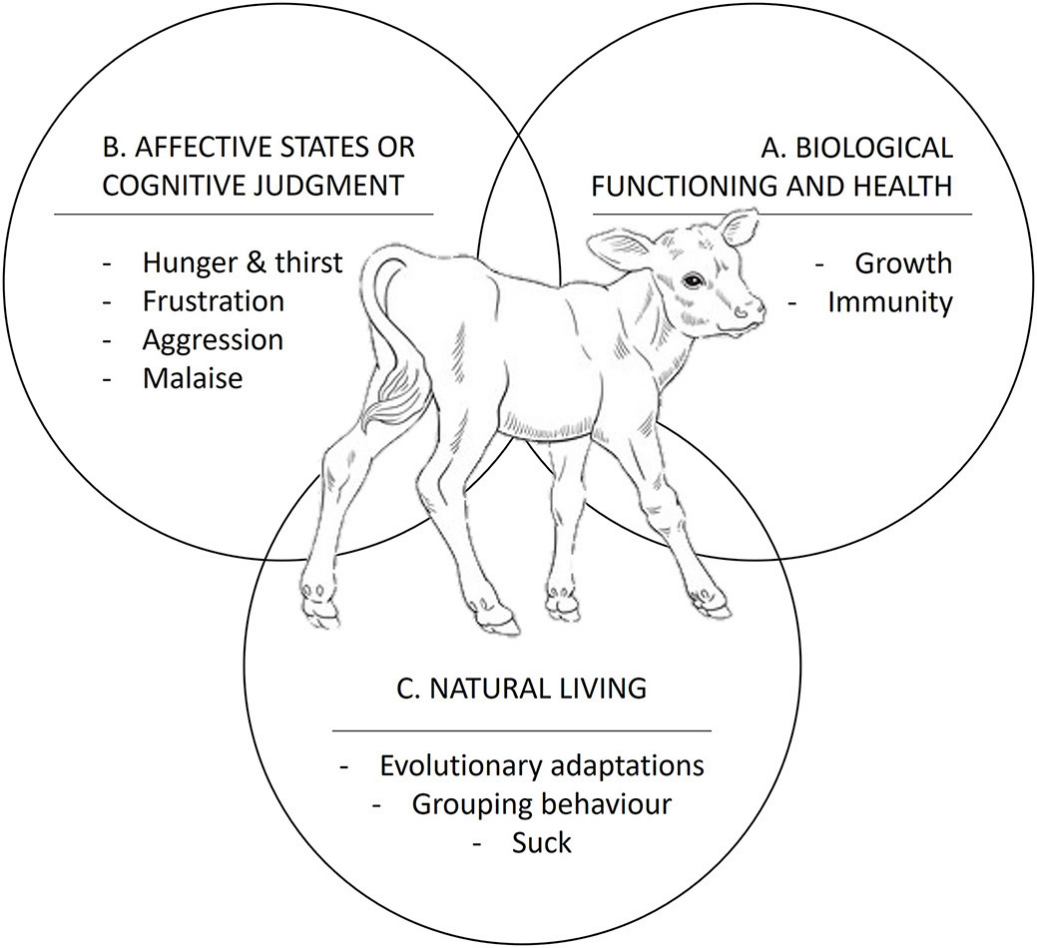
The three spheres of animal welfare and corresponding keywords: **(A)** biological functioning and animal health, **(B)** affective states or cognitive judgement, and **(C)** natural living (Adapted from 9).

It is therefore essential to define animal welfare before evaluating any management strategy. Animal welfare indicators can be grouped into three basic concepts (represented by spheres) first defined by Fraser et al. (7) and later adapted for dairy cattle by von Keyserlingk et al. (8). The three key spheres are (i) biological functioning and health, where good health indicates the correct physiological functioning of the animal; (ii) affective states or cognitive judgement, which considers how the animal feels when experiencing and perceiving its environment (8); and (iii) natural life, which refers to the evolutionary adaptation suffered of the animal to its environment, such as gregarious behavior (9) (Figure 1).

Ensuring optimal welfare in all three spheres during the rearing period has a direct impact on calf development. This is important because it has been shown that optimal development during the early stages of an animal's life influences its future (10), for example, neonatal diarrhea and other neonatal parameters have an economic cost and are associated with adverse effects on future cow production and reproductive performance (11, 12). This concept implies that rearing a healthy calf up to puberty under the highest welfare conditions will result in optimal production in future lactations (13). Growth rate during the first 6 months of life has been shown to be a direct determinant of age at first calving (14). In addition, body weight at first calving is associated with higher milk yield in the first lactation (15). Therefore, the efficiency of the dairy system can be improved through optimal calf rearing, a lower age at first calving, optimized future performance (16, 17), reduced rearing costs and shorter non-productive periods.

Dairy calves are highly susceptible to stress throughout their rearing period, but the most critical period is before weaning. There are many stressors during the pre-weaning period. The first stressor is the separation from the mother (18) and the potentially negative effects of human-animal interaction (19). Later in the animal's life, transport to a new location (20) and other management practices, such as pain during the disbudding (21), discomfort due to suboptimal housing conditions, and the limited opportunities for social interaction with their conspecifics (22), can also affect animal welfare. In addition, dietary management is key to the proper physiological and immune development of the animal (23). However, it is necessary to examine the interpretation of this management from an animal welfare perspective.

Significant changes in calf management have occurred over the last few decades, and many different realities have coexisted (24, 25) due to the diversity of production systems around the world. Despite the existence of some calf rearing guidelines such as FAO (26) and NASEM (27), there is little research on how management or the lack of an appropriate management affects welfare. Farm management strategies need to accurately identify, target, and intervene when different calf stressors occur. Focusing on feeding programs (16, 28) and social management (20) are high potential strategies that farmers can implement to avoid welfare problems. It is also important to address the lack of standardized and universal good management practices related to the welfare of dairy calves.

However, there is an unclear protocol available in the literature to ensure the highest welfare from feeding and social management strategies for preweaned dairy calves to have a base on which all realities can be established. In addition to studying how each of the management strategies affect the three spheres of animal welfare. Furthermore, no literature review has been undertaken to examine all these issues.

For these reasons, the first part of this systematic review was undertaken to systematically map the research in feeding management strategies and identify any existing scientific gaps in knowledge. This work is also intended to prioritize actions and future research, as well as exploring the interpretive approach to this management. However, such a comprehensive review is lacking in the current state of knowledge. The following research question was formulated: What is known from the literature about the feeding management of preweaned calves and how does it affect welfare? What needs to be investigated?

## 2. Materials and methods

A systematic review was used to address our research objectives. The literature search was conducted according to the PRISMA guidelines (29). PRISMA stands for Preferred Reporting Items for Systematic Reviews and Meta-Analyses. These guidelines provide an evidence-based minimum set of items for the methodology and identification of publications and reporting in this review.

### 2.1. Search terms and search strategies

As a first step, the authors discussed the objectives of the search and the inclusion/exclusion criteria. It was decided to identify preweaned calves' feeding and social rearing strategies and to analyze their impact on the three welfare domains. Other management issues, such as disbudding, transport, or veterinary treatment, as well as more specific issues, such as milk composition or osmolarity, were not investigated. The search included literature published between the years 1975 and 2022. Only studies published in English and with a full scientific text available were included.

The search terms were defined using the PICO approach (population, intervention, comparison, and outcome) (30), modified for the study objectives (Table 1).

**Table 1:** Approach and structured steps used to search the literature for this review.

	**Three spheres of animal welfare**		
	**Biological functioning and health**	**Affective states or cognitive judgment**	**Natural living**
Population	Dairy OR calf OR calve^*^		
Intervention	farm^*^ OR wean^*^ OR rear^*^ OR “milk feed^*^” OR starter Or colostrum OR additi^*^ OR “solid feed^*^”	“individual hous^*^” OR “pair hous^*^” OR “milk bucket” OR bottle^*^ OR deprivat^*^ OR enrich^*^	“early separat^*^” OR “pair hous^*^” OR mother OR separat^*^ OR “milk bucket” OR bottle^*^ OR “social group^*^” OR “social environment^*^” OR nipple
Comparison	health OR disease^*^ OR infecti^*^ OR disorder^*^ OR mortality OR longevity OR liveability OR pathogen^*^ OR phatologic^*^ OR cull^*^ OR metabolic^*^ OR perform^*^ OR “body condition^*^” OR develop^*^ OR immun^*^ OR environment OR ruminat^*^ OR rumen^*^	behavio^*^ OR stereotyp^*^ OR environment^*^ OR “fear test” OR “open field” OR “novel object test” OR “restrain test” OR “behavio test^*^”	behavio^*^ OR stereotyp^*^ OR environment^*^ OD “maternal bond^*^”
Outcome	perform^*^ OR feed OR milk OR consumption OR intak^*^ OR starter OR “body weight” OR weaning^*^ OR OR growth OR “early digest^*^” OR APPs OR cortisol	fear OR hunger OR learning OR stress OR cortisol OR aggressi^*^ OR optimist^*^ OR possitiv^*^ OR react^*^ OR upset^*^ OR cognit^*^ OR judg^*^ OR pain^*^ OR mal^*^ OR discomfort^*^ OR thirst^*^ OR anxiet^*^ OR affect^*^	behavio^*^ OR “social interact^*^” OR activ^*^ OR “social buffer^*^” OR explorat^*^ OR aggressi^*^ OR upset^*^ OR playful^*^ OR suckling^*^ OR adapt^*^ OR group^*^ OR greg^*^ OR play^*^ OR rest^*^ OR voc^*^

### 2.2. Data extraction and search process

The searches were performed on May 27th, 2021. The defined search terms resulted in two databases in Pubmed and Scopus, which yielded 984 publications (Pubmed) and 697 (Scopus). This means that the search identified 1,681 publications as potentially relevant. An update of the search was performed on July 26th, 2022, just before the manuscript was finalized, using the same search terms but restricting the search to the period after the original searches were performed, thus including literature between May 27th, 2021, and July 26th, 2022, and yielding 102 new results.

**Figure 2:**
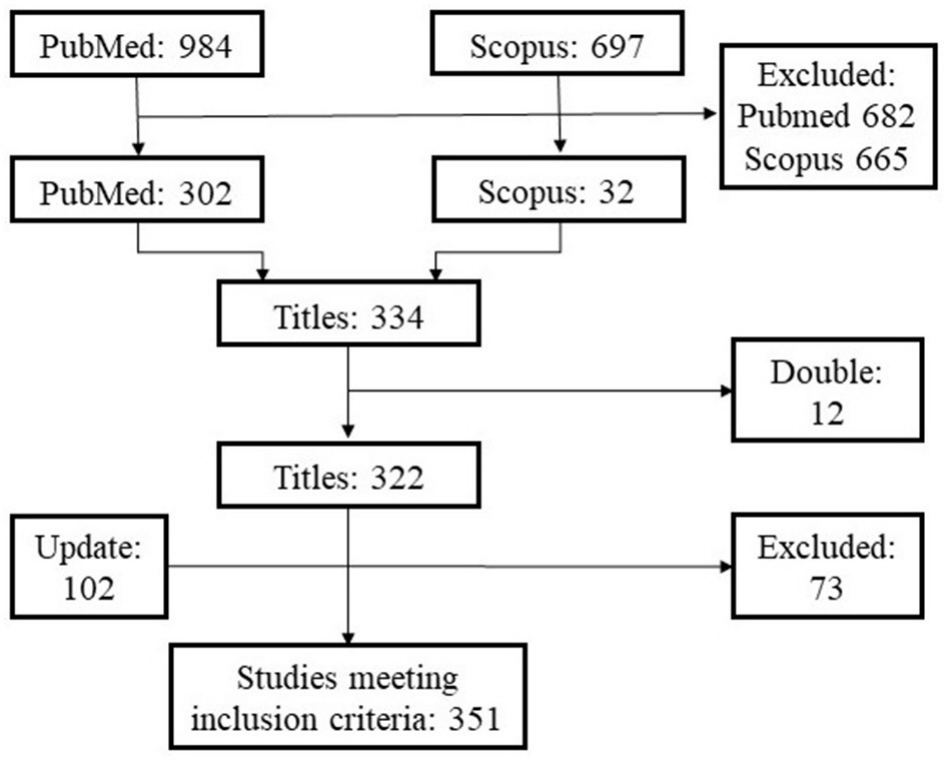
Search process for identifying publications on feeding and social management strategies in calf rearing.

After the initial search, the publications were scanned in several steps (see Figure 2). The papers were transferred to Abstrackr (31), a web application that facilitates the screening of systematic reviews by title and abstract. The publications considered relevant in terms of management or welfare issues in each of the Abstrackr filters were combined, resulting in a single dataset of 334 publications. These studies were included in a database with title, authors, journal, year of publication and DOI.

The same person (first author) filtered all the papers, and each author double-checked for each 25% of the papers. In this study, the level of agreement between the authors was 86.3%, with 80% or more being strong agreement, as reported elsewhere (32).

The updated search identified an additional 102 publications. Only 29 of the new articles were considered as relevant. The 80.2% were excluded throughout the review as they did not meet the inclusion criteria. Abstracts were removed if they did not relate to the welfare, feeding, or social management strategies of dairy calves.

After screening the titles and abstracts, the search results were refined using the screening tool “Rayyan” (33), where duplicates were removed and 322 publications were relevant to be included as results of the systematic review search. Each of the remaining publications was examined by reading the abstract and categorized according to animal welfare sphere and management resources. For animal welfare, publications were grouped into three spheres of biological functioning and health, affective states or cognitive judgement and natural living. As there is an interrelationship between the spheres, when publications addressed welfare from more than one area, they were included in the corresponding groups. Clustering was done according to: colostrum, milk replacer, started feed, weaning, mother bonding, social interaction, and human interaction. After a full reading of the abstract, a complete reading was performed to sort into the correct category if this information was unclear. After updating and screening, 351 studies met the inclusion criteria, as shown in Figure 2.

## 3. Results

### 3.1. Study characteristics

**Figure 3:**
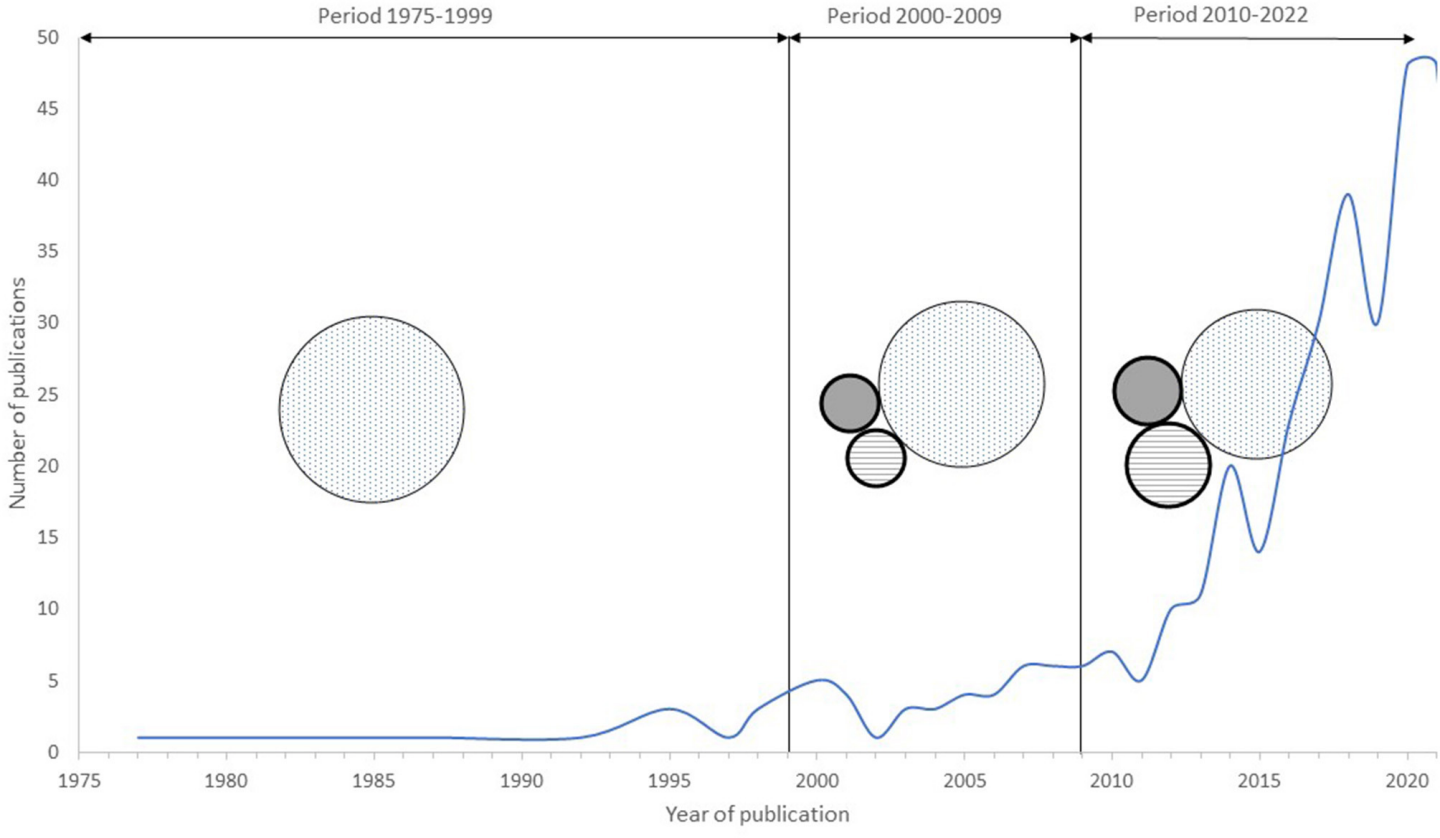
Distribution of publications according to the year of publication and three spheres of animal welfare. The blue line represents the number of publications per year related to animal welfare during the rearing of Holstein's calves. The size of spheres is weighted by the percentage of publications related to each animal welfare sphere. Dotted spheres represent biological functioning and health, spheres with horizontal lines refer to natural living, and gray spheres refer to affective states or cognitive judgements.

Based on the available scientific publications, there has been a noticeable upward trend since 1975 which continues up to the present day, with 82% of the publications having been published in the last 10 years (62% of which have been published in the last 5 years). The scientific research can be broadly grouped roughly under three broad, interrelated headings of welfare: 68.1% relates to biological functioning and health, 18.9% to natural living, and 13% to affective states or cognitive judgement. However, publications with the last two major components were published very recently, in the last decade. From 1975 to 2000, all the publications were related to biological functioning and health. In 2001–2010, 80.5% corresponded to biological functioning and health, 9.7% to natural living, and 9.8% to affective states or cognitive judgment. In particular, in the last interval from 2011 to 2022, 65.2% of the publications covered biological functioning and health, 20.9% natural living, and 13.9% affective states or cognitive judgment (Figure 3).

The 351 studies were published in 50 journals representing 49% of the Journal of Dairy Science articles. Preventive Veterinary Medicine represents 5.9%, Animals 5.4%, Journal of Dairy Research and PLoS One 3.7% each, and Frontiers in Veterinary Science 1.7%. The remaining 30.6% is spread over 44 other journals.

**Figure 4:**
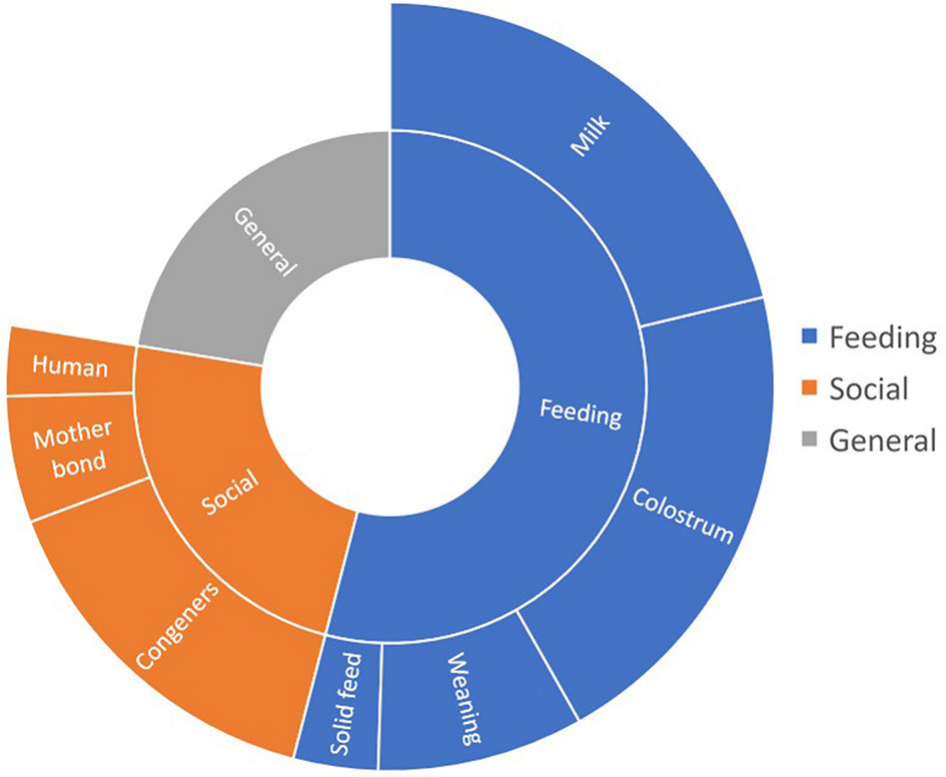
Distribution of papers by topic of publications when searching for feeding and social management strategies. The size of each is proportional to the number of publications found.

In this first part, we analyze all the feeding management techniques and their impact on welfare. According to the specific topic addressed, the publications can be classified, from most to least number, into general management (22.5%), milk replacers (20.5%), colostrum (19.7%), social interactions (16.9%), weaning (8.8%), mother bond (5.4%), started feed (3.4%) and human-animal relationship (2.8%) (Figure 4). Although many topics were addressed, even when dealing only with management practice were considered, the studies could be divided into two main groups according to the nature of the practices: (i) feeding and (ii) social management. As these groups are so large, they are considered separately.

### 3.2. Synthesis of results of feeding management

**Figure 5:**
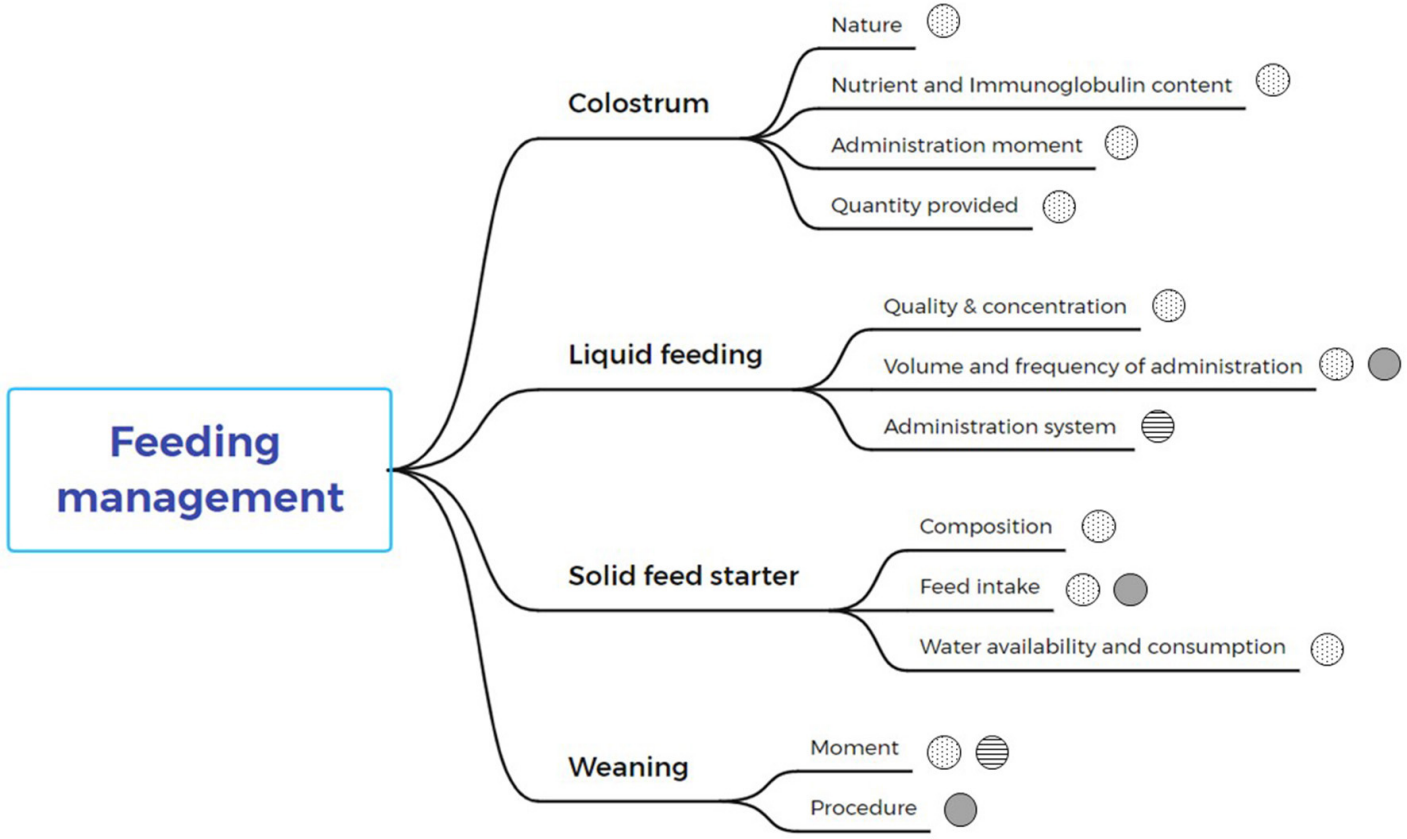
Critical points of each phase of feeding management are identified and their relationship to animal welfare spheres. Dotted spheres represent biological function and health, spheres with horizontal lines relate to natural living, and gray spheres relate to affective states or cognitive judgement.

Feeding in the early stages of calf life is critical for good development. Several studies have investigated the effect of feeding management techniques during the early stages of calf development, particularly in preweaned calves. Compared to the framework of the three spheres, all the different steps of feeding management have been studied in a compartmentalized manner. Under the umbrella of feeding management each component of colostrum, liquid feed, solid feed starter, and weaning strategies are evaluated and analyzed in Figure 5.

## 4. Discussion

Despite the importance of the neonatal and infant period for appropriate physical, behavioral, and cognitive development into adulthood (34), the literature review over the last two decades has produced many publications on different management strategies, but few studies from the perspective of the three welfare domains.

However, there has been a shift in the approach to animal welfare assessment, incorporating animal-based indicators related to affective states and natural living. The application of this new welfare knowledge will improve the daily lives of animals.

### 4.1. Feeding management for welfare

Feeding management during the first period of calf life is crucial to ensure their development, welfare, and productivity (23). The effect of feeding strategies on the development of preweaned calves has been reported in several of the papers reviewed in this analysis. In addition, the effect of each feeding management practice on animal welfare has been investigated in several publications. Thus, as shown above, in Figure 5, the key aspects of each feeding management are explained from the (a) correct colostrum administration (35), (b) liquid feeding until weaning (36, 37) (c) feeding with solid starter feeding (38, 39) and (d) weaning management (40).

#### 4.1.1. Colostrum management

According to the studies reviewed, colostrum intake affects welfare from a biological function and health perspective, as it is essential for the immunity of the calf. Publications have shown that it is crucial to provide sufficient quantities of high-quality colostrum with nutritional and immunoglobulin content and to achieve this immunity in the 1st h of life. High-quality colostrum has an IgG concentration >50 g/L (41). In the studies reviewed, two approaches were used to assess the impact of colostrum on calf immunity, colostrum characteristics and passive immunity assimilation in the calf (see Table 2) (50).

**Table 2:** Cut-off points for passive immunity transfer in calf serum according to total serum protein and IgG concentration from different review studies and the equivalence measured with a Brix refractometer.

	**Failure**	**Fair**	**Good**	**Excellent**	**Authors**
Total serum protein (g/l)	<52		>52		(42–44)
	<58–63		>58–63		(45)
IgG concentration (g/l)	<10				(46–48)
	<10	10–18	18–25	>25	(35, 49)
	<20–25	>20–25			(45)
Brix (%Brix)	<8.1%	8.1–8.8%	8.9–9.3%	>9.4%	(35)

In terms of colostrum characteristics, maternal and commercial substitutes have been studied as two types of colostrum (according to their nature). Commercial substitutes have adequate IgG absorption and are less likely to be microbiologically contaminated (51). However, when maternal colostrum is offered, calves show increased growth at weaning, improved immune and metabolic development, and higher of blood IgG concentrations (51–53). The quality of maternal colostrum varies depending on the individual cow and environmental management factors. For example, several studies have shown that multiparous cows produce better colostrum than younger cows, as it has a higher concentration of IgG and better nutritional properties (46, 54). However, a proper vaccination protocol and adequate dry cow feeding are essential to reduce passive transfer failure (54).

The time of collection and the time between collection and administration are also important. If the quality of the colostrum is poor or if it is administered at an inappropriate time, the transfer of passive immunity will fail. This leads to a decline in the wellbeing of the biological function and health. Therefore, the longer it takes to collect the colostrum after calving, the lower the IgG concentration will be (46), and its administration to the calf must be carried out in the shortest possible time (55).

If colostrum cannot be administered immediately, hygiene and storage practices are considered key factors. Under poor hygienic conditions, colostrum may be bacterially contaminated (50, 55). If it is not possible to maintain optimal hygiene, heat treatments such as pasteurization at 60° for 60 min (41, 50, 51) or high-pressure treatment at 400 MPa for 15 min (56) can be used. These treatments reduce the concentration of pathogenic bacteria and maintain IgG quality (56, 57). In addition, colostrum can be stored frozen as freezing and thawing do not affect IgG concentrations as long as thawing is performed au bain-marie and the temperature does not exceed 40°C (58).

It is also the key to assessing colostrum quality. The Brix refractometer is an accurate, acceptable, and rapid tool for assessing colostrum quality evaluation tool with excellent repeatability (59, 60). Accordingly, colostrum can be classified as good if >22% Brix and poor if <18% Brix (61). It is important to note that mixing poor quality colostrum with good quality colostrum is not recommended (62, 63). Although the quality of colostrum has been extensively studied, its relationship to the quantity to be administered has not been established. Therefore, increasing the amount of colostrum, reducing the time between birth and colostrum administration, or increasing the amount of whole milk after colostrum have been recognized as good practices (42, 62, 64) and improve welfare from a biological function and health perspective.

As mentioned above, the characteristics of the colostrum are as important as the immunity assimilation of the calf. The success or failure of passive immunity transfer has been extensively studied (35, 43, 46). For example, a relationship has been found between successful passive transfer and a lower likehood of developing enteric or respiratory disease has been found (65). In addition, lower concentrations of IgG and total serum protein in the first 3 days of life are associated with reduced growth rates (43, 46). Based on the literature reviewed, the cut-off values for transfer failure and the calf serum IgG concentration measured with a Brix refractometer are shown in the table below (Table 2).

All of the above mentioned assumes that good quality and quantity of colostrum is essential for calf rearing and to ensure welfare from a biological function and health perspective at this stage (42, 49, 66). In short, the best colostrum management protocol, with less passive transfer failure, is administer a volume of high-quality colostrum that is equivalent to 10–12% of their body weight in the first 2h and an additional meal corresponding to 5% of body weight 6–8 h later to reduce morbidity and mortality (67).

#### 4.1.2. Liquid feeding management

According to the studies reviewed, the management of liquid feeding affects animal welfare in all three spheres (23). From the point of view of biological function and health, it is essential to provide liquid feeding of good quality, concentration and volume so that the animal is well nourished. In addition, the amount of liquid feeding and the frequency of feeding will affect affective states or cognitive judgement, as calves properly fed should not suffer from hunger. The delivery system also affects the natural living sphere, as nipple-feeding is more similar to natural sucking behavior.

Calves must be adequately fed to meet their nutritional requirements and to support the development and maturation of the gastrointestinal tract's, allowing the calf to digest and absorb nutrients (23, 38, 68). Insufficient milk intake slows postnatal growth and can affect the development of organs such as the intestines and the mammary glands (23). Liquid feed intake also influence solid feed intake (69) and calf growth. According to Soberon et al. (70), the higher the average daily gain during preweaning, the more milk will be produced in the first lactation. Epigenetic programming, which is still under investigation, suggests that diet is one of the most important environmental factors influencing the genetic expression of milk production (70). However, the optimal feeding strategies (38) are highly uncertain in the studies reviewed. In addition, adjustments to the management in calf feeding practices will inevitably be required. At this stage, different alternatives have been studied, taking into account the type of liquid diet and supplement, the amount and concentration, the frequency of administration or the method of administration. Regardless of the strategy adopted, correct implementation of hygiene is essential to prevent health problems in calves, reduce the burden of pathogenic bacteria and break the chains of infection. For example, there are several studies that focus primarily on the cleaning of artificial nipples and buckets, as these are presented as the central critical point (71).

On the other hand, no significant improvement in calf development was found in relation to milk type. The reviewed publications have focused on the use of a milk replacer, transition milk (72), or discarded milk (73). However, when using milk replacer, the most critical factor is to maintain a protein content above 28% (74, 75), as milk protein content is directly related to daily gain (76). Fat content must be maintained in the range of 17–25% (72, 74, 77). It is important that the milk replacer is of high quality, as poor-quality milk replacers can affect welfare through morbidity (diarrhea) and also hunger through starvation (78).

There is a wide variety of feeding protocols in the reviewed bibliography, and there is no consensus on the best practice. Traditionally, restricted feeding has been used to promote solid feed intake, but these restrictions have resulted in malnutrition and immunosuppression (38), contributing to a negative welfare status. In contrast, other authors have investigated *ad libitum* milking administration protocols, with growth benefits but delays in rumen development as animals consume less solid feed (79, 80). Therefore, a balance needs to be found between encouraging the calves to start eating solids and avoiding starvation if they are fed with milk only. Other protocols involving the amount, frequency or concentration of milk have also been reviewed. For example, feeding 20% of the calf's bodyweight in milk has been shown to reduce feed intake and rumen development before weaning (23, 81). Alternatively, rumen development is better at 10% of the bodyweight (81). In contrast, some protocols provide an amount of milk regardless of body weight, with varying amounts and concentrations, as shown in Table 3. The optimal number of dosed meals per day is not known (86).

**Table 3:** Quantities and concentrations of milk fed to calves, according to different studies.

**Quantity**	**Concentration (powdered milk)**	**Author**
6 L/d	750 g/d	(80)
5–9 L/d	–	(82)
4.4 L/d	660g/d	(77)
6–8 L/d	–	(83)
3.8 L/d 3.8, 5.6, 7.2 L/d	454 g/d 454, 681, 908 g/d	(84)
4.7 L/d	660 g/d until 39 d 330 g/d since 42 d	(52)
9L/d during 3–28 d 5l/d during 29–42 d	941 g/d 778 g/d	(85)

Several authors have pointed out that the feeding protocol has a significant impact on welfare. Depending on the protocol, the calves may suffer from hunger or frustration, which would worsen animal welfare at affective states or cognitive judgment and natural living spheres. In order to know whether the animals are hungry, non-nutritive oral behaviors (87, 88), cross-sucking (89) and vocalizations (90, 91) could be studied. In addition, when animals do not feel hungry, they engage in more locomotor play, which is a positive indicator of welfare (82, 85). Despite the lack of a clear protocol on the amount, concentration, and frequency of administration in the review results, several authors have reported better results in terms of health and growth outcomes with fixed amounts of liquid feed at higher nutrient densities throughout the lactation period compared to a gradual increase (74, 83, 84).

Regardless of the protocol used, there are several ways to offer milk. Bucket feeding is far removed from the natural sucking behavior of the animal, and teaching animals to drink from a bucket requires training and effort. Up to 60% of calves know how to drink milk from a bucket at 3 days of age t (92). Another option is to use bottles with nipples, which are more compatible with the natural living sphere. With this method, animals show less non-nutritive sucking (88). In addition, throughout the literature reviewed, the method of feeding has been modernized with the introduction of automatic milk feeders, which are introduced to animals at around 5 days of age and can be housed in groups of 10–15 calves (93, 94). These feeders accurately control animal milk intake (95), but their effect on calf welfare is still being investigated.

For all of the above, the authors emphasize the need to provide good quality milk and choose an appropriate feeding protocol, with a fixed amount of milk offered at the beginning and gradually reduced as weaning approaches, to meet the calves' nutritional needs of the while avoiding hunger (38, 96). It is also important to monitor animal behavior to know if they are hungry if there is an increase in vocalizations or non-nutritive oral behaviors. Further research is needed to know the best amount, concentration, and frequency that ensure the best animal welfare in the three spheres.

#### 4.1.3. Solid feed starter management

The literature reviewed shows that solid feed management has a significant impact on growth and welfare. At the level of affective states or cognitive judgement, correct feed management helps to reduce hunger or digestive discomfort. Diet composition, intake and water availability are essential for ruminal development, and therefore affect animal welfare through the biological function and health sphere.

Proper rumen development during the preweaning is critical. Solid feed intake plays a fundamental role in rumen development and maturation. The milk feeding protocol has a major influence on solid feed intake, and high liquid diet feeding programmes may compromise solid feed intake in the first few weeks of life (38, 68, 69). The most important factor in promoting solid feed intake is the decrease in milk available after 40 days of age, as this can lead to malnutrition before this time (96). In addition, social contact, which will be discussed in more detail in Part Two, also appears to influence intake, with social animals consuming more solid starter feeds (97).

A solid diet should provide the protein and energy necessary for calf growth (an average of 23.4% protein and 32.3% starch on a dry matter basis) (39, 98). In addition, the method of feeding, the palatability of the solid food, and the amount consumed are also important for the calf growth and the avoidance of digestive distress (98) which would reduce the welfare at the level of affective states or cognitive judgement.

In addition to starter feed, calf feeding practices should include the provision of water *ad libitum* to maximize starter intake and weight gain. Weight gain is reduced when animals are deprived of water (99), and animal welfare deteriorates (21).

On the other hand, it is currently debated whether the inclusion of forage in the starter diet can benefit calves (39). Forage feeding has been promoted from a welfare perspective. Some authors report benefits such as alleviation of ruminal acidosis, promotion of ruminal microbial diversity and abundance (100) as well as higher average daily gain. The importance of feeding hay not only for rumen development, but also for reducing stress during the weaning process (101). Others have found negative effects of including hay, such as a reduction in solid starter consumption (39), which is crucial because when calves have consumed enough starter, it is time to wean them (102).

#### 4.1.4. Weaning management

Weaning has also been the subject of much research, as it is a turning point in the intensive calf feeding management and can cause a great deal of stress. Weaning is a very stressful event for the animals and a challenge for the farmer (103, 104). It also affects animal welfare at the level of biological functioning and health as it radically changes the diet and the calves need to have a proper rumen development. From the point of view of affective states or cognitive judgement, the procedure used to carry out weaning can cause anxiety and frustration. Finally, at the level of natural living, this event causes behavioral changes in the calves.

Weaning is the most important nutritional transition for young calves. On intensive dairy farms, calves are weaned earlier than in the wild, where weaning occurs at around 6 months (104). In the studies reviewed, it was found that the timing of weaning can be decided the basis of two main parameters in order to minimize adverse effects. Either it can be programmed according to the age of the animal or the amount of solid starter food consumed (105). In addition, weaning can be managed gradually (removal of feed), by diluting the milk, or abruptly by removing access to liquid feed (106).

As explained above, milk restriction is commonly used to encourage solid food intake to facilitate early weaning, but it can compromise calf growth if done too early (107). The earliest age at which this procedure can be done is 40 days, as it can cause malnutrition if done earlier (96). In all the studies reviewed (103, 108, 109), this weaning is carried out up to 62 days.

In addition, solid feed intake is considered the key parameter in deciding when to wean calves, and it has been suggested that calves are ready for weaning when they have consumed a minimum quantity of 0.9–1 kg of solid feed for three consecutive days (102) or 15 kg of cumulative non-fiber carbohydrates (52). The problem with deciding when to wean an animal using this method is that many calves are weaned at an older age than if age had been the deciding factor, and very individualized management is required (105).

The weaning protocol has also been widely discussed, and each strategy has a different effect (Table 4). Gradual weaning is carried out by removing meals. This encourages a greater consumption of solid feed and helps to develop the rumen better (96, 104, 109). It is the most similar to natural weaning (111), although it has been shown to cause a more prolonged frustration in the animal. In contrast, abrupt weaning removes meals all at once and causes less frustration (103). However, some animals may be unwilling to consume the minimum amount of solid feed, especially if they are on *ad libitum* milk allowances (109). Finally, the last option is to dilute the milk replacer until only water remains, and then remove the nipple, which causes minor frustration (110).

**Table 4:** Weaning strategies and their effects that each of them has on the calf, according to the different authors.

**Weaning strategy**	**Effect**	**Authors**
Wean for age	Easier farm management	(96, 107)
Wean for solid feed intake	Ensured ruminal development	(102, 105)
Abruptly weaning	High stress Not accustomed to eating solid feed	(103)
Gradual weaning	Higher feed consumption Less abnormal behavior	(96) (109)
Greatest underlying frustration	(103)
Dilute weaning	Less frustration	(110)

However, regardless of how weaning is performed, it is a stressful process for calves (i.e., the daily gain decreases the day after weaning, and calves have high cortisol concentrations (112). It is known that calves increase the frequency of vocalizations during this period, a measure of stress and distress (90, 104), but there is still a lack of knowledge on how to minimize the stress suffered during this period. However, the effects of this process on affective states or cognitive judgement have not been investigated.

Weaning management is therefore important as it must be carried out to avoid decreasing nutrient intake and weight loss. Best management practices show a gradual reduction in milk offered from 40 days of age and complete weaning when they consume more than 1 kg of feed for three consecutive days.

## 5. Conclusions

Calf welfare is not sufficiently considered when making management adjustments. There are still many common calf feeding management practices applied, paricularly in the dairy farm sector, that are detrimental to the health and welfare of calves. Understanding the welfare problems caused by management and the consequences of not doing so, will help to prevent future problems. A standardized protocol helps to have a basis on which to build on according to different production systems. The most studied issues are colostrum and solid feed starter management. However, with all the information reviewed, the most important gaps in knowledge are the lack of a clear protocol for administering milk replacers to reduce hunger and the best management of weaning to reduce stress. Collaboration between the scientific research community and the dairy sector is essential to establish management standards and ensure the success of farm systems adaptated to support proper growth, ensure health and welfare, and facilitate weaning.

## 6. Implications

This paper provides an overview of the feeding management strategies used in the rearing of Holstein calves and how this management affects the three spheres of animal welfare. Understanding the influence of management on welfare helps to prevent future problems. From the information reviewed, the best protocol, according to the authors, is detailed below. In addition, the authors have produced a table (Supplementary Table 1), suggesting different management practices and their impact on each of the spheres of animal welfare and the missing gaps that need to be investigated in the future.

Based on the information reviewed, some advice could be summarized to optimize calf management protocols in terms of feeding management.

The most important aspect of colostrum management is to collect and administer it as soon as possible after birth, in the first 2h. If possible, pasteurize it to minimize the microbial load. Calves should drink a high-quality colostrum with a minimum of 22° Brix, and a good volume corresponding to 10–12% of their body weight. With regard to liquid feeding, it is essential to provide a high-quality milk substitute (>28% protein, 17–25% fat in powdered milk) and optimal hygiene. A fixed amount of 6–7 liters with a minimum of 660 g of milk powder in two or three daily feeds is recommended. The solid feed starter should provide the protein and energy needed for calf growth, and the animals must have continuous access to water. It is important to facilitate an increase in the rate of feed intake during the first few weeks of age to promote the correct rumen development of the calf. Finally, the best protocol for weaning is to gradually reduce the amount of milk offered from 40 days of age and to wean completely when calves consume more than 1 kg of feed for three days.

## Data availability statement

The raw data supporting the conclusions of this article will be made available by the authors, without undue reservation.

## Author contributions

PC, AV, FE, and IB-P contributed to the conception and design of the study. PC and IB-P organized the database. PC drafted the first manuscript. All authors contributed to the revision of the manuscript, read, and approved the submitted version.
