# Supplementary material for: Welfare implications on management strategies for rearing dairy calves: A systematic review. Part 1–feeding management
Source: Front Vet Sci. 2023 Mar 30;10:1148823. doi: 10.3389/fvets.2023.1148823 (PMC10150452; doi:10.3389/fvets.2023.1148823)
Supplement: Supplementary file 1 [file Table_1.docx]

Table S.1. Relationship between the different management practices and their impact on each animal welfare sphere. The symbol “-“ indicates to a lack of information on how the management practice affects welfare. The symbol “!” indicates missing gaps that need to be investigated in the future.

|  |  | Three spheres of animal welfare | | |
| --- | --- | --- | --- | --- |
|  | Practice management | Biological functioning and health | Affective states or cognitive judgment | Natural living |
| C  O  L  O  S  T  R  U  M | Administer maternal colostrum | Acquire passive immunity (higher immunoglobulins) | - | Same that receive in nature |
|  | Administer commercial colostrum | Acquire passive immunity (immunoglobulins) | - | - |
|  | Nutrient and Ig content | Important for adequate immunity | - | - |
|  | Administration moment | Shortest possible time after delivery | - | - |
|  | Assimilation of passive immunity | Good level of IgG and TP in calves’ serum | ! | - |
| M  I  L  K | Type, quality, and concentration | Correct development and maturation | Not feel hungry | ! |
|  | Volume and frequency | Well feed | Not feel hungry or frustration | Ad libitum is the same that nature |
|  | Administration system: nipple, bucket or automatic feeders | Automatic allows controlling individually | ! | Nipple more natural sucking behaviour |
| S  O  L  I  D  F  E  E  D | Composition | Ruminal development | Reduce digestive discomfort | ! |
|  | Feed intake | Ruminal development | Reduce hunger | ! |
|  | Water availability | Influences on feed intake | - | - |
| W  E  A  N  I  N  G | Wean for age | From 40 days of age | ! | Earlier than wild |
|  | Wean for solid feed intake | Better rumen development | Less stress | ! |
|  | Abruptly weaning | Not accustomed to eating solid feed | High stress  ! | Increased vocalizations |
|  | Gradual weaning | Better rumen development | Frustration  Less stress | Increased vocalizations |
|  | Dilute weaning | Better rumen development | Less frustration | ! |
